# Human-Centered Design for Public Health Innovation: Codesigning a Multicomponent Intervention to Support Youth Across the HIV Care Continuum in Mozambique

**DOI:** 10.9745/GHSP-D-21-00664

**Published:** 2022-04-28

**Authors:** Trena I. Mukherjee, Allison Zerbe, Joanna Falcao, Shauna Carey, Alexandra Iaccarino, Brynn Kolada, Bruno Olmedo, Cady Shadwick, Hitesh Singhal, Lauren Weinstein, Mirriah Vitale, Eduarda De Pimentel De Gusmao, Elaine J. Abrams

**Affiliations:** a Department of Epidemiology, Columbia Mailman School of Public Health, New York, NY, USA.; b ICAP at Columbia University Mailman School of Public Health, New York, NY, USA.; c ICAP at Columbia University, Maputo, Mozambique.; d IDEO.org, New York, NY, USA.; e Department of Pediatrics, Vagelos College of Physicians and Surgeons, Columbia University, New York, NY, USA.

## Abstract

Adolescents and young people represent a growing proportion of people living with HIV (AYAHIV), and there is an urgent need to design, implement, and test interventions that retain AYAHIV in care. Using a human-centered design (HCD) approach, we codesigned CombinADO, an intervention to promote HIV viral suppression and improve antiretroviral therapy (ART) adherence and retention in care among AYAHIV in Nampula, Mozambique. The HCD process involves formative design research with AYAHIV, health care providers, parents/caretakers, and experts in adolescent HIV; synthesis of findings to generate action-oriented insights; ideation and prototyping of intervention components; and a pilot study to assess feasibility, acceptability, and uptake of intervention components.

CombinADO promotes ART adherence and retention in care by fostering peer connectedness and belonging, providing accessible medical knowledge, demystifying and destigmatizing HIV, and cultivating a sense of hope among AYAHIV. Successful prototypes included a media campaign to reduce HIV stigma and increase medical literacy; a toolkit to help providers communicate and address the unique needs of AYAHIV clients; peer-support groups to improve medical literacy, empower youth, and provide positive role models for people living with HIV; support groups for parents/caregivers; and discreet pill containers to promote adherence outside the home. In the next phase, the effectiveness of CombinaADO on retention in care, ART adherence, and viral suppression will be evaluated using a cluster-randomized control trial.

We demonstrate the utility of using HCD to cocreate a multicomponent intervention to retain AYAHIV in care. We also discuss how the HCD methodology enriches participatory methods and community engagement. This is then illustrated by the youth-driven intervention development of CombinADO by fostering youth empowerment, addressing power imbalances between youth and adult stakeholders, and ensuring that language and content remain adolescent friendly.

## Abstract (precis)

Using a human-centered design approach, we codesigned CombinADO, an intervention to improve antiretroviral therapy adherence and retention in care among adolescents and young people living with HIV (AYAHIV) in Nampula, Mozambique. CombinADO aims to foster peer connectedness and belonging, provide accessible medical knowledge, demystify and destigmatize HIV, and cultivate a sense of hope among AYAHIV.

## Teaser

**Key Findings:**

- Using human-centered design (HCD), key learnings identified opportunities for intervention to promote viral suppression and improve ART adherence and retention in care among adolescents and young people living with HIV (AYAHIV). Opportunities included fostering a sense of belonging and peer connectedness with other AYAHIV, providing contextually resonant medical knowledge that fits the unique context of adolescence and young adulthood, reducing misconceptions and stigma surrounding HIV, and cultivating a sense of hope for AYAHIV.
- Intervention components include a media campaign to reduce HIV stigma and increase medical literacy; a toolkit to help providers communicate and address the unique needs of AYAHIV clients; peer-support groups to improve medical literacy, empower youth, and provide positive role models for people living with HIV; support groups for parents/caregivers; and discreet pill containers to promote adherence outside the home.

**Key Implications:**

- Public health practitioners should consider how HCD provides a pathway for public health innovation, shortens the research to practice gap, engages marginalized communities, and integrates novel interventions into existing complex systems when coupled with other synergistic research methods such as implementation science.

Resumo em português no final do artigo.

## INTRODUCTION

HIV is the leading cause of death among adolescents and young adults in sub-Saharan Africa, and the second leading cause of death globally.^1^ Mozambique is among the 10 countries with the highest HIV prevalence (12.6%) globally, and among the 6 countries that account for more than half the population of adolescents living with HIV (ALHIV).^2^^,^^3^ With nearly two-thirds of the population under age 25 years, and nearly half (47%) under age 15 years,^4^ Mozambique is facing a substantial youth bulge.

There are an estimated 140,000 adolescents (aged 10–19 years) and 320,000 young people (aged 15–24 years) living with HIV (AYAHIV) in Mozambique, and AYAHIV remain a vulnerable population.^5^ Although nearly 70% of ALHIV globally acquired HIV infection through vertical transmission,^6^ adolescents and young people are at greater risk for acquiring HIV due to multiple and intersecting forms of discrimination and structural inequality, lack of access to age-appropriate health services, high rates of poverty, unemployment, and illiteracy.^7^ Poor HIV and sexual health knowledge contribute to disproportionate rates of HIV in Mozambique, where only 30% of adolescent boys and girls know how to prevent HIV.^3^ For adolescent girls and young women, early sexual debut and high unmet need for family planning contribute to poor health outcomes, and 3 of every 5 girls aged 15–19 years in Nampula, Mozambique have had at least 1 pregnancy.^8^ Despite recent progress, viral suppression among AYAHIV, particularly adolescent boys and young men, remains lower than adults aged 25 years and older and is negatively correlated with age. Among AYAHIV aged 10–14 years, 15–19 years, and 20–24 years, viral suppression is 64%, 72%, and 80% among females, respectively, and 60%, 62%, and 75% among males, respectively.^4^

To achieve the UNAIDS 95-95-95 goals, an integrated approach across the HIV care continuum is needed for AYAHIV. Health systems are dynamic, resource constrained, and interconnected. For this reason, complex health interventions that address multiple, intersecting factors at the individual, interpersonal, community, and societal levels and that adapt to local contexts are essential for addressing the HIV epidemic.^9^ Prior evidence suggests using a combination approach that consists of biomedical, structural, and behavioral interventions is best suited for addressing AYAHIV.^10^ A systematic review of recent (i.e., those published between 2015–2019) interventions to improve antiretroviral therapy (ART) adherence among AYAHIV in lower- and middle-income countries identified only 7 interventions.^11^ The 3 interventions at the individual level were not found to improve ART adherence and viral suppression. Of the 4 interventions at the community level, the 2 interventions that included home-based visits from community-based support workers or peer counselors significantly improved ART adherence. Thus, interventions designed to target a single barrier to ART adherence are insufficient, and a combination of interventions is warranted. To maximize uptake of a combination of interventions, the dynamic nature of contextual factors must be taken into account across the individual, community, and policy contexts.^12^ Therefore, the integration of interventions into existing practice must be considered early in the process of program development and implementation. Furthermore, acknowledging that AYAHIV are not a homogenous population and that they are influenced by peers, family, and their community, tailored approaches are crucial to meet the unique challenges, strengths, and opportunities that adolescents and young people possess.^10^^,^^13^

Based on prior evidence of interventions for AYAHIV, we conclude that interventions that target a single barrier to ART adherence are insufficient.

Human-centered design (HCD) is a person-based approach for social innovation that is well-suited for developing interventions in partnership with youth.^14^ The HCD approach helps shape interventions that are grounded in the needs and desires of AYAHIV and other stakeholders across the health system and optimizes the likelihood that interventions will be integrated into existing health systems.^15^ Initially developed in the private sector, HCD seeks to develop services and products based on a holistic understanding of consumer needs, aspirations, and behaviors and has been successfully employed by the private sector to develop products such as the retail experience at the Apple store and Oral-B toothbrushes.^16^ More recently, HCD has been employed by the public sector to develop programs, services, or products for social innovation.^17^ The U.S. President's Emergency Plan for AIDS Relief (PEPFAR), the U.S. Agency for International Development (USAID), and their implementing partners have used HCD to promote HIV prevention and reproductive health globally. Examples include pre-exposure prophylaxis (PrEP) rollout in Zambia;^18^^,^^19^ integrating sexual and reproductive health and HIV testing and prevention^20^ with promoting ART adherence for adolescent girls and young women in Rwanda;^21^ improving TB preventative treatment uptake among people living with HIV in Kenya;^22^ and promoting HIV service uptake among men who have sex with men in the Philippines.^23^

Although HCD is increasingly being employed to develop public health services and programs, it remains a novel approach to intervention development and dissemination in global health research.^15^^,^^24^ In this article, we introduced HCD as an approach that enriched participatory methods and fostered community engagement when developing complex, multicomponent interventions. HCD methodology is then illustrated by the CombinADO study, which aimed to promote HIV viral suppression and improve ART adherence and retention in care among AYAHIV in Nampula, Mozambique. Finally, we conclude with a discussion of the advantages of using an HCD approach to cocreate acceptable, feasible, and sustainable interventions and how this method is particularly well suited for youth populations.

## METHODS

### What Is Human-Centered Design (HCD)?

HCD is a creative problem-solving approach for developing, refining, and testing products, programs, and services.^16^ This approach is adaptive and flexible by employing a structured, interactive approach for engaging stakeholders (e.g., adolescents, people living with HIV, health care workers [HCWs], and policy makers) in rapid iteration cycles that diverge (create choices) and converge (make choices) until a concept is determined to be viable, feasible, and desirable by key stakeholders to ensure that interventions are both wanted and sustainable.

HCD uses a structured and interactive approach to engage stakeholders in rapid iteration cycles until a concept is deemed viable, feasible, and desirable by key stakeholders.

Through the application of design thinking, HCD seeks to understand the diverse dynamic interconnections within a complex system to understand barriers and facilitators of implementation and scale-up. While HCD offers an approach to inciting empathy to define and understand the problem and to designing and evaluating solutions, design thinking is the iterative process that leads to the development of solutions that are viable, feasible, and desirable.

HCD also facilitates interdisciplinary collaboration among stakeholders with diverse backgrounds^25^ that may include individuals with subject matter and/or design expertise in a variety of fields such as sociology, public health, systems science, systems or service design, visual or graphic design, and design research. As a participatory approach, HCD also facilitates empowerment and collective ownership by integrating community members into the research team. As active members of the research team, communities assist with identifying appropriate research questions, data collection, interpretation of results, intervention development, adaptation, and implementation strategies. The ability to empathize is crucial for HCD and allows researchers to set aside their own assumptions and gain real insight into the needs, wants, and aspirations of the communities for whom they are developing interventions. This allows communities to confront the issues that impact them most, address community resistance up front, incorporate community values to generate buy-in, and build consensus early on.

**FIGURE 1:**
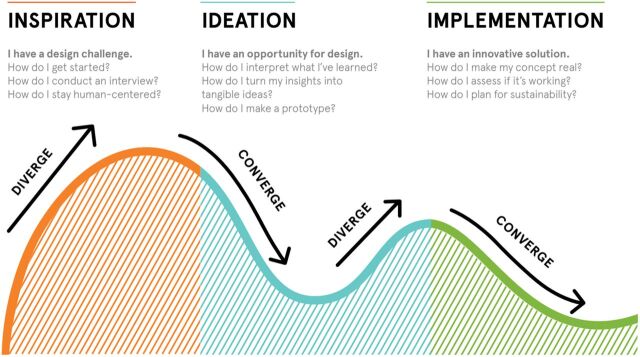
What Is Human-Centered Design?

The HCD approach and design thinking processes can vary by organization.^24^ However, HCD consists of distinct phases in which the problem is first identified and defined (i.e., inspiration); early concepts are generated, tested, and refined (i.e., ideation); and solutions are integrated into the larger system (i.e., implementation) (Figure 1).^24^^,^^26^ Figure 1 and Box 1 provide an overview of HCD and the design thinking process that the design firm IDEO.org used for this project.^26^ Although displayed linearly, it is important to note that design thinking may be cyclical, and it is not uncommon to circle back to previous steps.^25^

BOX 1 **IDEO.org's Five Steps for Design Thinking**

**Design Research Defines the Problem**

Design research involves contextual mapping and formative research to identify a community's needs, wants, desires, and constraints. The goal of design research is to understand communities, their influences, and obtain a wide range of experiences using a rapid and flexible process. Although quantitative data may be collected, qualitative data using ethnographic or anthropological methods are most often used to allow for the researcher to be more embedded within the community and to learn through observations. Data capture involves rapid field notes, photos, videos, or artifacts (e.g., products purchased from a local market or drug store) to enable rich storytelling through visual or media output. Data are then transferred to sticky notes, in which 1 key finding, quote, or image is placed onto a sticky for data management. Although unconventional in traditional research, sticky notes allow for data that are concise and distilled into its simplest form to enable quick written or visual data sharing.

**Insights Identify Opportunities for Intervention**

In the next phase, synthesis involves rapid, flexible, and iterative analysis of design research findings to generate actionable insights and to identify areas for intervention (“design opportunities”). The use of sticky notes allows for rapid data processing as data are grouped and regrouped to identify themes. Themes that emerge from design research are distilled into succinct statements that summarize the most valuable research findings (“insights”). Synthesis frameworks can be applied to inform intervention development by eliciting empathy and using visual storytelling to communicate messages to place findings in context and understand the human experience of the user.

**Ideation Brainstorms Potential Solutions to Problem**

Ideation starts with generating open-ended “how might we” statements in response to the insights developed during synthesis. These statements reframe the problem as an opportunity to elicit a large number of ideas for innovative solutions. Once the ideation session is complete, the ideas are consolidated, refined, and short-listed based on feasibility, desirability, and viability constraints.

**Prototyping Allows Rapid Hypothesis Testing**

Unique to human-centered design is the process of prototyping individual components of an intervention, with the goal of developing a combination intervention that intersects desirability, feasibility, and sustainability upon scale-up. Prototyping enables hypothesis testing, challenges underlying assumptions, and facilitates learning from small scale, intentional “failures” in a controlled setting. Through rapid cycles of iterative cocreation, feedback is gathered from individuals, their peers, and influencers. Prototypes are then iterated and improved upon in the next cycle until an optimal intervention is developed for larger-scale testing.

**Implementation Scales Up Sustainable Solutions**

Once desirable, feasible, and sustainable prototypes are developed, they are ready to be combined and evaluated in a small-scale pilot. During implementation, the intervention is integrated into the larger system, and all products, services, and branding are finalized and scaled up. The final intervention is then monitored and evaluated through various study designs (e.g., randomized control trial).

### Development of the CombinADO Intervention

**FIGURE 2:**
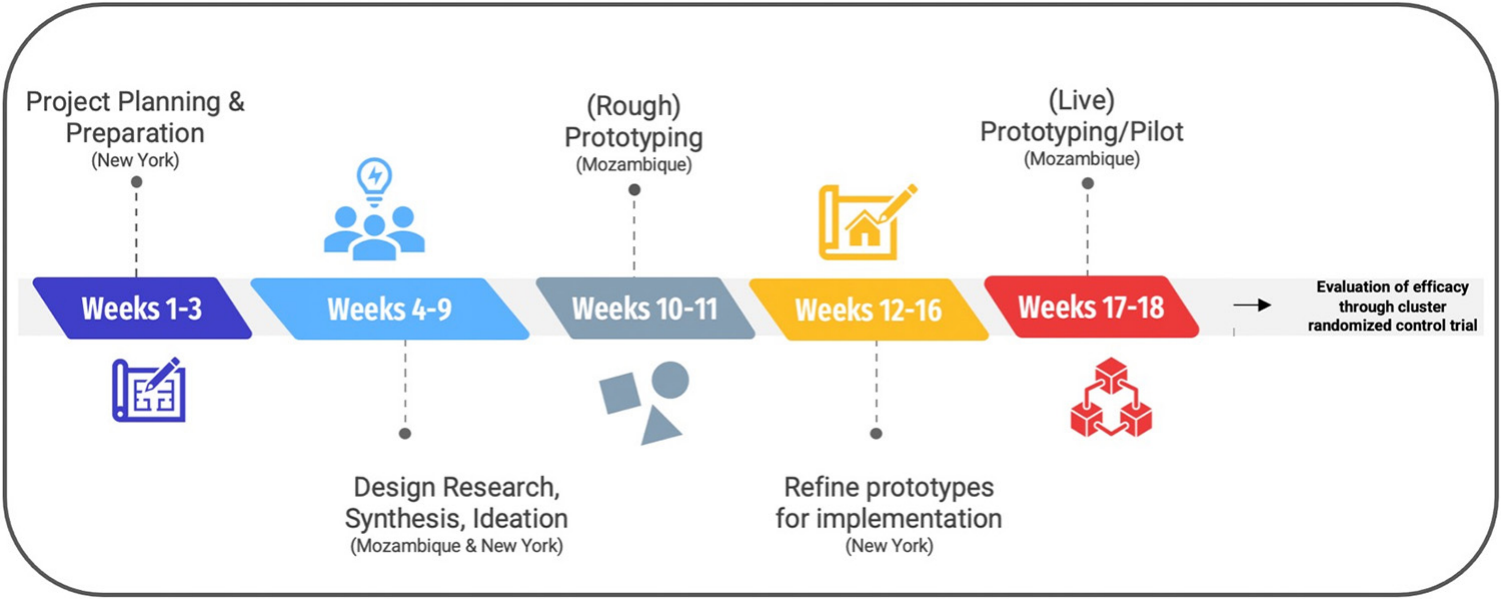
Timeline of Human-Centered Design Approach to Developing the CombinADO Intervention in Mozambique

In 2018, the National Institute of Child Health and Development put out a request for research proposals to generate much-needed scientific innovation to yield effective public health interventions for young people affected by HIV in resource-limited settings Prevention and Treatment through a Comprehensive Care Continuum for HIV-affected Adolescents in Resource Constrained Settings (PATC^3^H).^27^ Researchers from ICAP at Columbia University, in collaboration with the Mozambique Ministry of Health, were awarded a grant to use HCD to complete formative research to design (Phase 1, Figure 2) and evaluate (Phase 2) a combination intervention to improve HIV-related health outcomes among AYAHIV in Nampula, Mozambique. This article solely focuses on intervention development, which was completed in Phase 1.

To develop the intervention, research investigators collaborated with IDEO.org, a nonprofit organization that specializes in partnering with organizations and using HCD methodology to develop and scale products and services for social innovation. To develop a multicomponent, clinic-based intervention to improve uptake of ART among young people living with HIV, design researchers from IDEO.org and research investigators from ICAP and the Mozambique Ministry of Health attended several in-person meetings in New York to develop a research plan and protocol, followed by 16 weeks of data collection and intervention prototyping in Nampula (Figure 2).

### Study Participants

**FIGURE 3:**
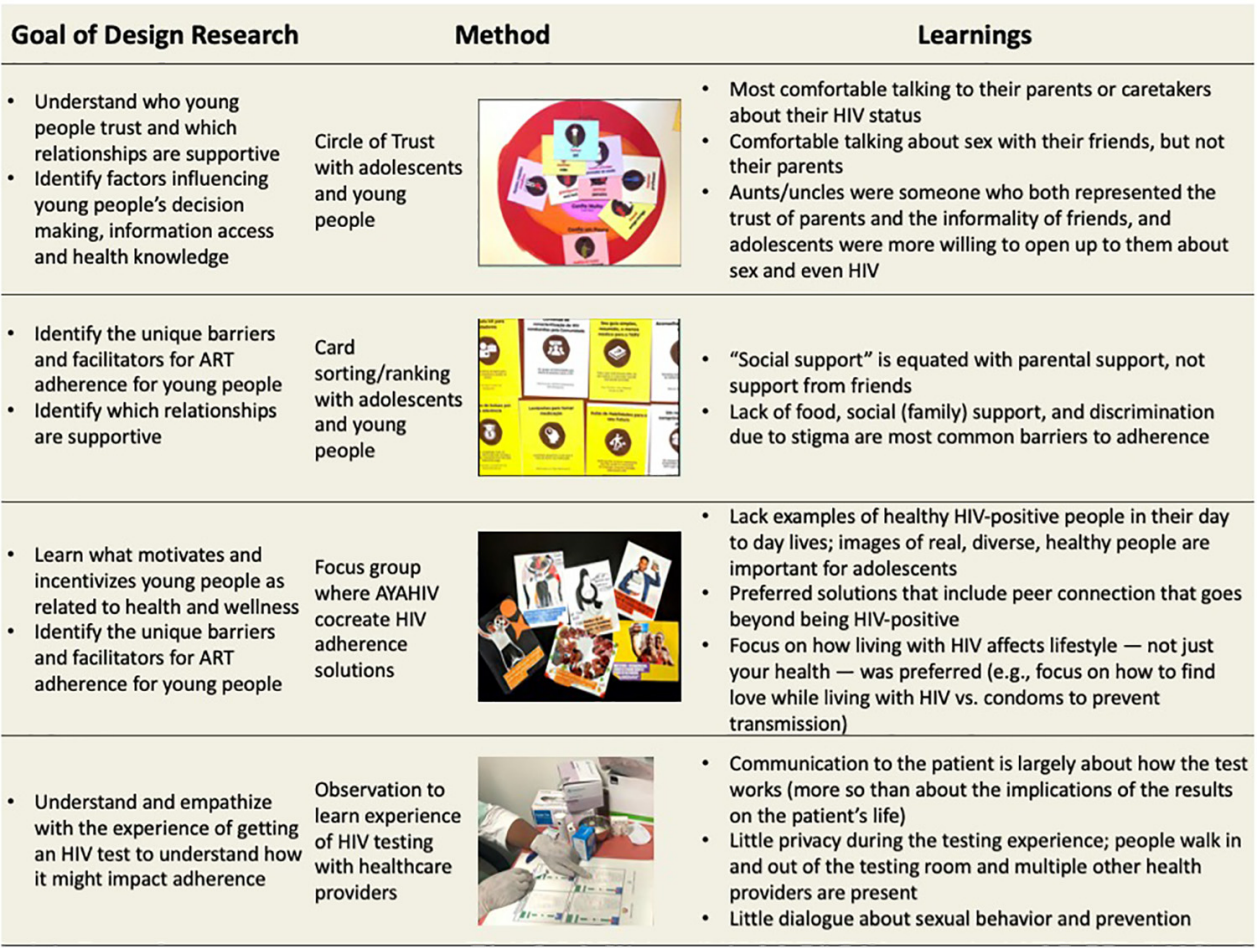
Design Research Findings With Key Stakeholders on ART Adherence for AYAHIV in Mozambique^a^ Abbreviations: ART, antiretroviral therapy; AYAHIV, adolescents and young people living with HIV. ^a^More details about each method can be found at: https://www.designkit.org/methods.

We purposively recruited seropositive and seronegative adolescents (aged 15–18 years; n=4) and young people (aged 19–24 years; n=19), parents of AYAHIV (n=6), key community influencers (n=7), health care providers (n=5), and national and international youth HIV experts (n=7) in Nampula, Mozambique. Purposive sampling is typical for HCD as the goal is to understand the environment and context rather than the generalizability of findings. Participants were excluded if they did not fit the group profile or were unable to provide assent/consent. Study activities varied based on participant preference, profile, availability during study activities, and scheduling constraints (Figure 3). Participants were compensated 300 MZN (US$5) for their time and transportation.

### Ethics Approval

This study was approved by ethical review boards at Columbia University Medical Center and the Mozambique National Bio-Ethics Committee for Health in July 2019. All adult participants provided written consent, and all youth participants provided written assent and obtained parent/guardian consent before engaging in any research activities.

## RESULTS

We provide an overview of the HCD approach employed by IDEO.org (Box 1), followed by our findings.

### Design Research

The goals of design research were to: (1) learn what motivates and incentivizes young people as related to health and wellness; (2) identify the unique barriers and facilitators for ART adherence for young people; (3) understand who young people trust, which relationships are supportive, and learn how they obtain health information; and (4) identify factors that influence young people's decision making, information access, and health knowledge.

Design research was completed with a total of 51 stakeholders over 4 weeks: 26 young adults aged 19–24 years, 4 adolescents aged 15–18 years, 9 key stakeholders, 6 parents of AYAHIV, 5 health care providers, and 1 young social media influencer in Nampula, Mozambique. Methods included ranking and card sort activities to understand who adolescents and young people trusted in a variety of health-related situations, identify the most common barriers and motivators for ART adherence, and confirm or refine hypotheses. Local translators and artists assisted with data collection and created materials used throughout design research (e.g., interactive data collection tools; Figure 3). Synthesis of design research findings to identify the learnings outlined in Figure 3 was completed over 4 weeks. Synthesis was primarily led by IDEO.org and the ICAP study team and was refined and clarified for language and content by AYAHIV using the methodology outlined in Box 1.

Design research revealed that adolescents were most comfortable talking to their parents or caretakers about their HIV status. In contrast, adolescents were comfortable talking about sex with their friends but not their parents. Aunts/uncles were someone who both represented the trust of parents and the informality of friends, and adolescents were more willing to open up to them about sex and even HIV. Participants cited lack of food, lack of social (family) support, and discrimination due to stigma as the most common barriers to adherence. Interestingly, social support is equated with parents and not friends. Participants recognized that solutions must include the community as well as the individual, and adolescents preferred holistic, motivation-based approaches (e.g., a sports league, mental health support, and classes to develop their professional skills) in addition to things like medication reminders. Staying healthy was the primary motivation for ART adherence, but adherence in the present was generally not associated with success and achievement in the future.

Design research participants cited lack of food, lack of social support, and discrimination due to stigma as the most common barriers to adherence.

The research team also completed several informal observations of health services to be able to empathize with adolescents and young people in Nampula. Through this, they learned that there is little privacy during HIV testing and little dialogue about the implications of the test result or about sexual behavior and prevention moving forward. The adolescent support groups missed the opportunity to empower youth to engage in peer support. Time spent with AYAHIV revealed how HIV interferes and hinders their lifestyle and a lack of representation of healthy AYAHIV. Messaging regarding how HIV affects lifestyle (not just health), how to achieve success, and how to form peer connections resonated the most with AYAHIV.

Finally, key informant interviews with experts in the field of adolescent HIV (e.g., ICAP project staff, health facility directors, and staff from regional health offices and the Ministry of Health) indicated that there was a lack of understanding of why prior programs had failed and that future programming must be adaptable, responsive to evolving client needs, and considerate of local culture to be successful.

### Insights

Synthesis led to several insights at the individual, interpersonal, and community levels. These insights were developed with the emotional experience across the HIV care continuum (i.e., journey mapping) and behavioral adherence archetypes (i.e., personas) in mind.

We developed insights with the emotional experience across the HIV care continuum.

#### Individual

- Drug fatigue isn't just about pills—it's about giving up on the future.
- People are motivated by what ART can do for their lifestyle, not their cells.

#### Interpersonal

- People will sacrifice their health to protect their secret.
- Young people have to continually “relearn” how to live with HIV at every big life milestone.
- Caregivers are the most important but often least prepared source of support for unmarried teens.
- The potential consequences of disclosure keep married people from their most powerful adherence asset: their partners.

#### Community

- In the uncertainty of poverty, people will trade tomorrow for today.
- Finding community amidst the loneliness of secrecy makes a lifelong journey feel possible.
- Contextually incompatible medical information causes gaps in ART adherence.
- Many people believe “If I don't feel sick, I don't need meds.”
- Where culture and science clash, people find confidence in compromise (i.e., the use of traditional cultural practices and Western medicine [ART]).
- When it comes to HIV, seeing is believing.
- Traditional gender roles inhibit men's access to HIV testing and treatment.

### Ideation

Eight “how might we” statements (Box 2) inspired idea generation of hundreds of potential solutions. Solutions were then sorted, ranked, and narrowed down into testable concepts based on accessibility, contextual relevance, and feasibility and were mapped out across the user journey and personas developed during synthesis. IDEO.org led ideation sessions in partnership with research staff from ICAP. AYAHIV and caregivers participated in cocreation workshops to further refine concepts and messaging.

BOX 2 **How Might We Support Youth Across the HIV Care Continuum?**

1. *How might we* redesign the testing moment to equip patients with the right information and motivation to engage with and adhere to their treatment?
2. *How might we* leverage checkups to reengage and troubleshoot beyond just measuring viral load?
3. *How might we* alleviate poverty-related barriers so that all young people have a chance to thrive on antiretroviral therapy (ART)?
4. *How might we* strengthen people's motivation to adhere to ART to go beyond “staying healthy”?
5. *How might we* help people living with HIV find a community that helps them thrive?
6. *How might we* support the trust circles of people living with HIV to support them and their treatment journey in the best possible way?
7. *How might we* destigmatize and normalize the mainstream narrative around HIV to increase retention and adherence?
8. *How might we* protect and enable people's discretion to facilitate their retention and adherence?

### Prototyping

**FIGURE 4:**
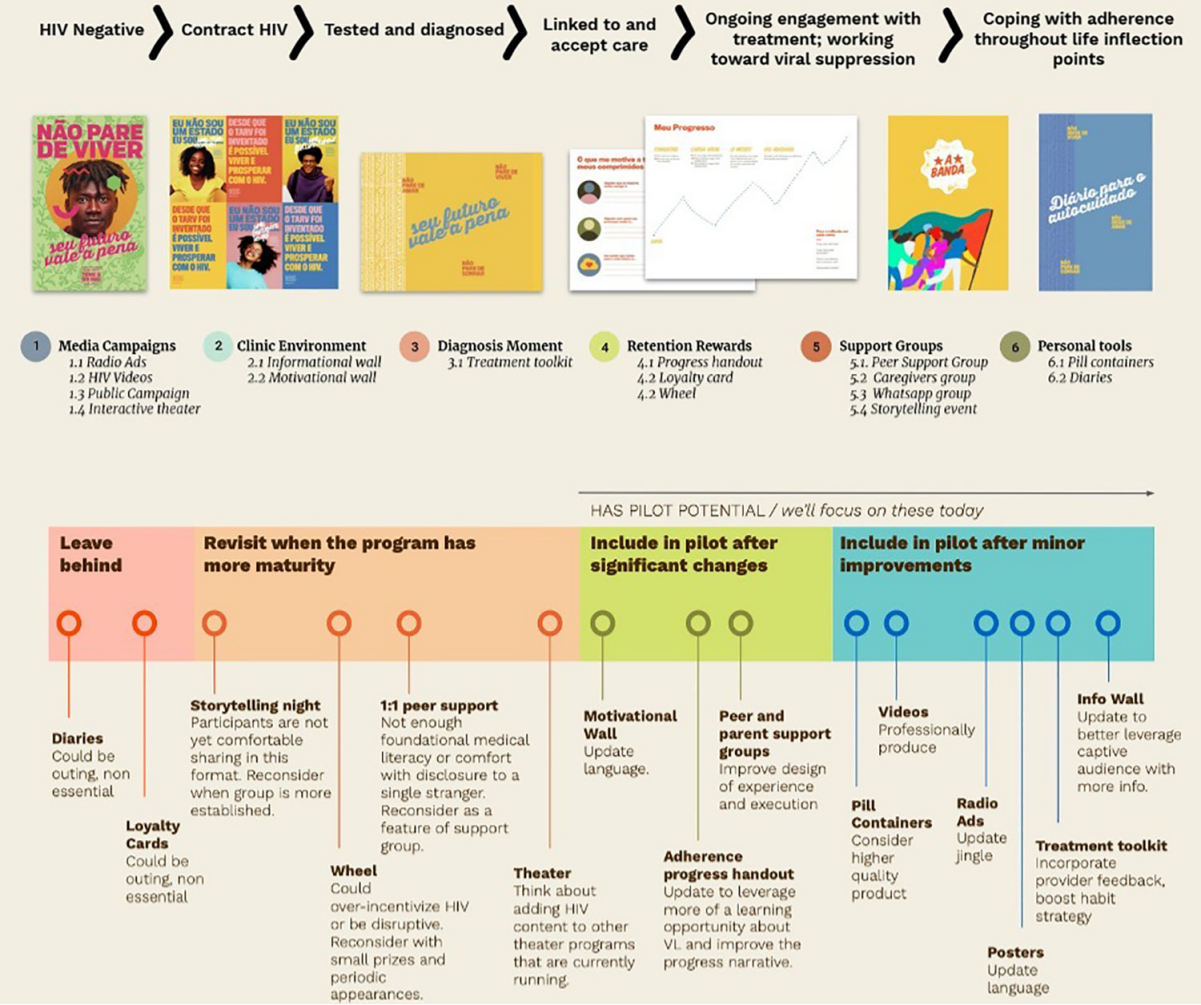
Prototyping Across the HIV Care Continuum

**FIGURE 5:**
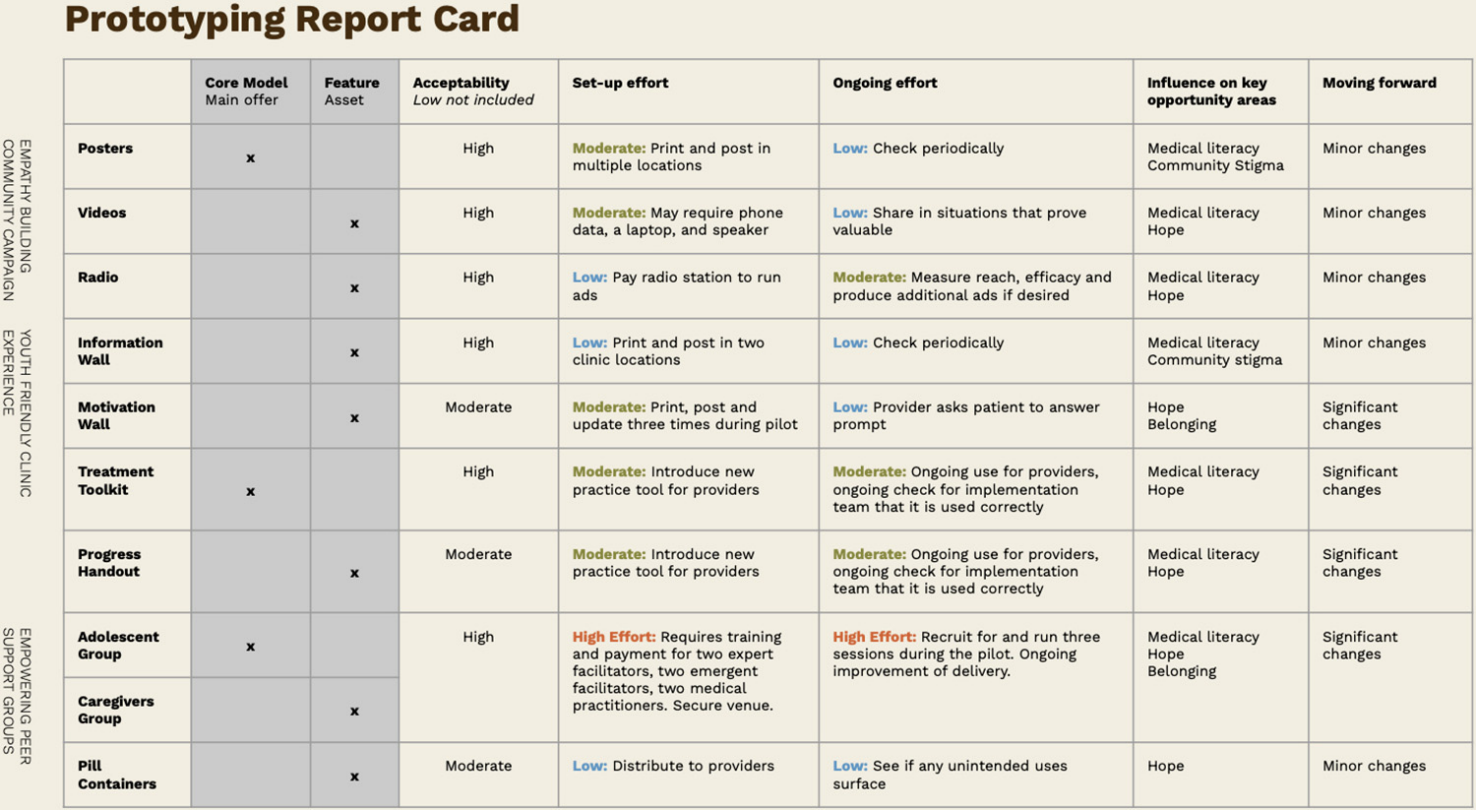
Prototyping: Lessons Learned

AYAHIV and health care providers cocreated prototypes alongside local artists and the research team. Low fidelity, rough prototypes were tested with 99 individuals including AYAHIV (n=63), caretakers (n=8), health care providers (n=5), community members (HIV-positive or -negative) (n=18), HIV peer support group facilitators (n=2), and codesigners/translators (n=3) over 2 weeks. Prototypes were evaluated for acceptability, desirability, feasibility, ability to address barriers to ART adherence and impact on behavior change and adherence. Data captured during initial prototyping sessions included observational data of participants interacting with prototypes, qualitative feedback, and brief open-ended surveys. These data were then interpreted by the research team, and key learnings were summarized. Prototypes that were removed from consideration included a motivational diary, loyalty cards, storytelling nights, and 1:1 peer support due to privacy and disclosure concerns (Figure 4). Ultimately, 12 prototypes spanning the HIV care continuum were selected; core components included support groups, media campaigns, and HIV treatment toolkits. Each component was then rated by the research team (Figure 5) based on acceptability, feasibility (set up and ongoing effort), influence on key barriers identified through design research, and changes needed in the next round of prototyping.

**Figure:**
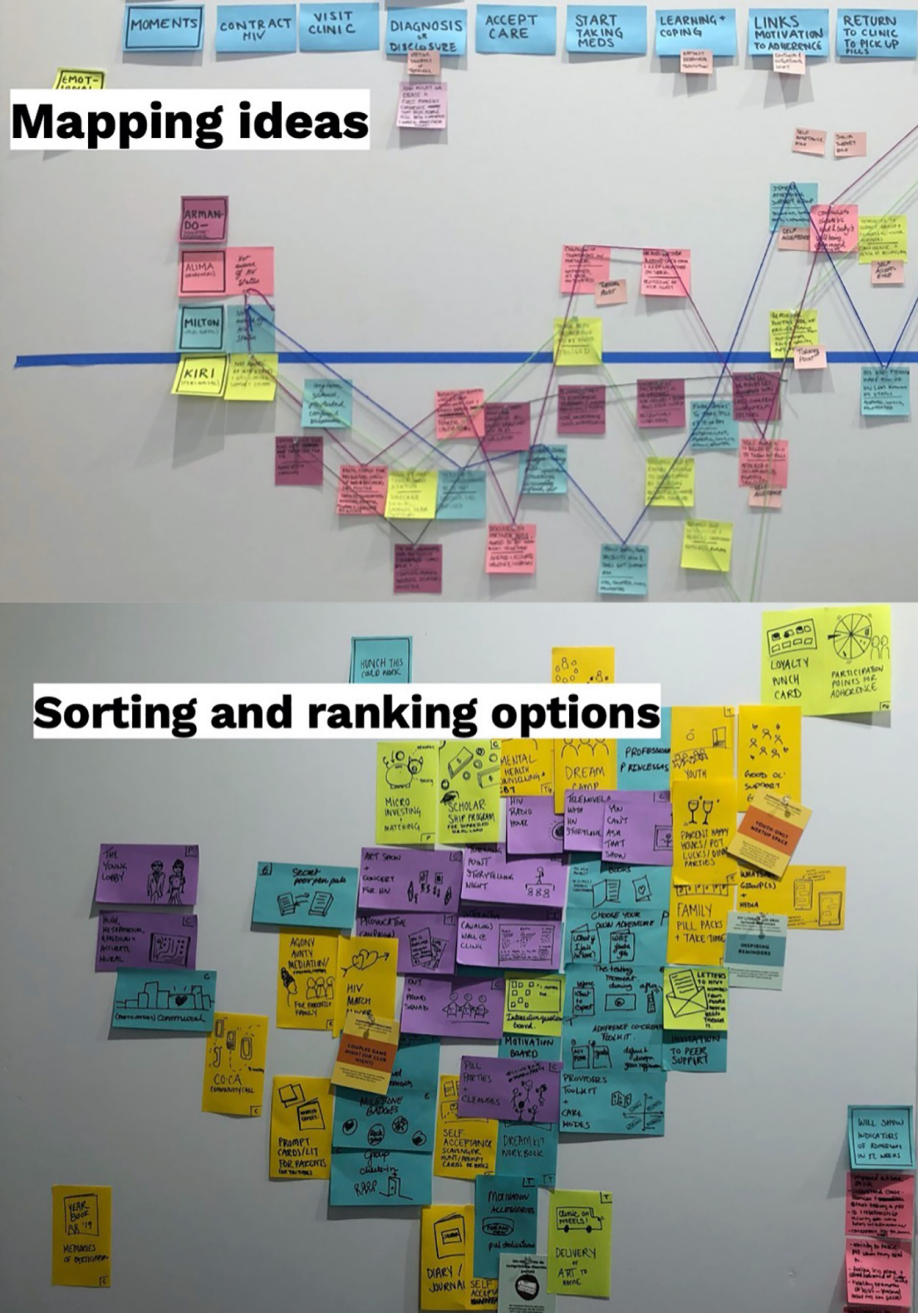
The top panel displays the application of synthesis frameworks (user journey by personas). The bottom panel displays the process of narrowing down concepts after ideation. ©2019 IDEO.org

Prototypes were iterated and improved upon or removed based on key learnings related to barriers and facilitators of ART adherence. Key learnings included that there is a culture of secrecy and fear surrounding HIV that prevents young people and their caregivers from connecting with other people living with HIV. However, AYAHIV can foster a sense of belonging, build confidence, and provide healthy examples of people living with HIV by placing ART adherence within the context of their daily lives. Moreover, providing contextually relevant medical information enables young people to understand the benefits and consequences of ART, so that they are better equipped and motivated to make choices that are conducive to adherence. Prior insights highlighted the need for increased knowledge about ART that is culturally congruent. Finally, HIV stigma is a major barrier to adherence, which is impacted by social situations, relationships, and community perceptions of HIV. HIV knowledge and empathy are needed to demystify and destigmatize HIV at the community level and to encourage disclosure of HIV status to caregivers or partners, who offer powerful adherence support.

Key learnings included that there is a culture of secrecy and fear surrounding HIV that prevents young people and their caregivers from connecting with other people living with HIV.

During prototyping, positive feedback from community members included:

*HIV materials usually have drawings. These posters remind us that real people who are smiling and healthy have HIV too.* —AYAHIV

*I learned to give real support and how to respond if any child comes to me when they are being discriminated against because of HIV. I will hug her and give her a kiss.* —Community member, HIV status unknown

*I had no idea 1 in 10 people in Mozambique have HIV. People in my life might have it, or the people I work with at the market. Why do we discriminate against it when it's so common? I don't think I could discriminate against the people I love.* —Patient at a health clinic, HIV status unknown

**FIGURE 6:**
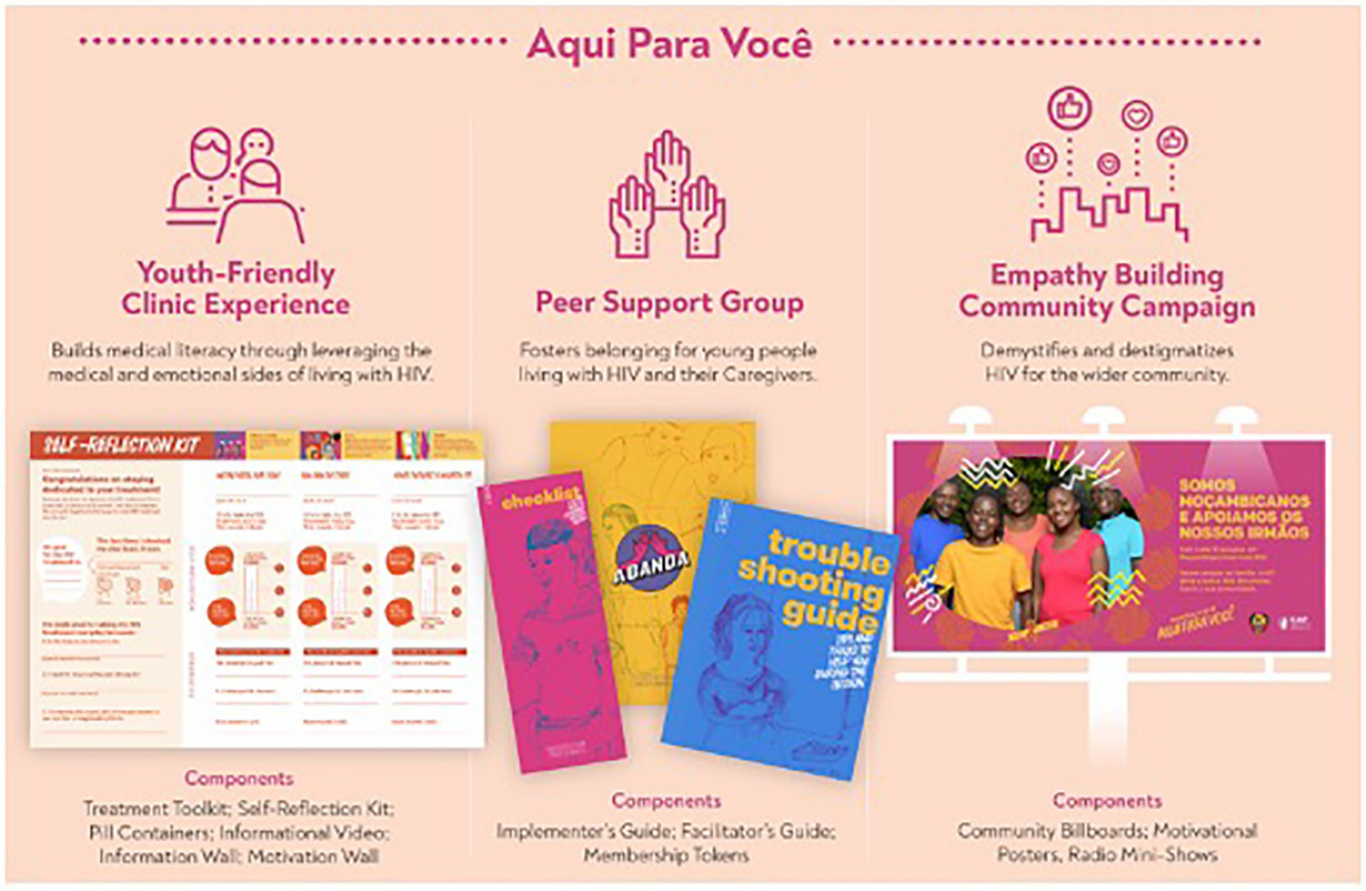
The Final Multicomponent CombinADO Intervention^a^ ^a^Peer-support groups unable to be piloted during coronavirus disease (COVID-19) pandemic.

The 12 prototypes (i.e., intervention components) were combined into a multicomponent intervention to be evaluated in a pilot study (Figure 5). Altogether, components were designed to promote adherence and retention in care by fostering peer connectedness and belonging (e.g., peer support); providing accessible medical knowledge (e.g., youth-friendly clinic experience); demystifying and destigmatizing HIV; and cultivating a sense of hope among AYAHIV (e.g., community empathy building) (Figure 6).

Each intervention component was then piloted at 2 urban health facilities in Nampula, Mozambique, over 12 weeks (July 2020–October 2020). Cross-sectional data were collected to evaluate acceptability, feasibility, and uptake of each component using direct observations (n=24) and exit interviews (n=74) after clinical consultations with AYAHIV; semistructured interviews with AYAHIV (n=42), HCWs (n=4), and key informants (n=2); focus group discussions (FGDs); and quantitative surveys with clients at each health facility (n=60). Descriptive statistics of quantitative survey data were completed in Stata.^28^ Interviews and FGDs were completed in Portuguese and then translated and transcribed. NVivo 12 was used to identify themes and emergent codes.^29^

HCWs recorded the total number of times they used the self-reflection kit, motivational wall, pillboxes, treatment tool kit, and informational wall daily. During the 12-week pilot, a total of 452 AYAHIV were seen across both health facilities. On average, the self-reflection kit was used 83% of the time, the motivational wall 91%, pillboxes 72%, and treatment tool kit 88%.

Direct observation of clinical consultations indicates that the pill containers were used 92% of the time, motivational wall 88%, self-reflection tool kit 79%, and the treatment tool kit 75%. Exit interviews with AYAHIV clients indicated that the majority used all CombinADO tools, with the treatment toolkit used the least (73%), and the video used the most (99%).

FGDs with the AYAHIV indicate that all prototypes were highly acceptable and appealed to clients of all ages and gender.

*These posters are more innovative; they are more up to date. There are certain posters at the health facility, for example about TB, where they do not use real people there. Now, these posters show real images, phrases, and motivational messages, and the people themselves who passed those messages. The messages are confirmed by the images.* —AYAHIV health facility client

In-depth interviews with the clients revealed that AYAHIV thought that the video and the treatment toolkits were the most useful tool to improve adherence and retention and minimize feelings of loneliness and stigma. More than 90% of AYAHIV who used the treatment toolkit and watched the video agreed that they had learned something new about ART and that these tools would help them with ART adherence. Themes drawn from in-depth interviews with AYAHIV included that these tools showed them that they can have a healthy family, that they feel that they are not alone, and that they were taught how to live with HIV.

*Because the [treatment toolkit] gives us answers to many things and the human being wants answers, he doesn't want doubts, the [treatment toolkit] gives these answers, and that helps, it makes us understand more what we are going through.* —AYAHIV health facility client

*I saw a lot of important things [in the video]—how we can protect ourselves, how we can get used to friends knowing that we have that disease, and many other things.* —AYAHIV health facility client

*The video came to teach me that people with HIV are not inferior to others, we are all the same and we can play, talk to anyone.* —AYAHIV health facility client

More than 90% of AYAHIV who used the treatment toolkit and watched the video agreed that they had learned something new about ART and that these tools would help them with ART adherence.

In-depth interviews with health care providers revealed that the video and treatment toolkit facilitated better communication with AYAHIV.

*With the video and the treatment toolkit, we have more contact with the teenager and there are certain questions that the [treatment toolkit] already has the answer to and he is already confident… and at some point, he becomes our friend. So, for me, it will help to retain them.* —Health care provider

One health care provider also described how the tools gave a renewed sense of hope for both themselves and their client once they understood how ART adherence and an undetectable viral load can fit into the context of a healthy life.

*I see teenagers who are already for 5 or 6 years taking ARVs but with high viral load, but why? [I ask them] “How many pills were left?… 5.” But now he has an objective for taking the pills and trying to be undetectable. So, I think this part helped me and I hope it helped them too.* —Health care provider

AYAHIV were also asked whether they understood all the information included in the tools and were assessed for HIV knowledge during exit interviews. Over 90% reported they understood the information presented to them across the tools; however, more than half (59%) were unsure about viral suppression and the concept of undetectable=untransmissible (U=U). This suggests that AYAHIV may need multiple opportunities to interact with the tools and communicate with their health care provider to improve ART health literacy.

In response to the coronavirus disease (COVID-19) pandemic, the Mozambique Ministry of Health recommended spacing HIV consultations 3 months apart, which meant that most AYAHIV included in the pilot only had 1 opportunity to interact with intervention components. This limited the ability to assess mid-term impact in areas where repetition may be especially beneficial for influencing outcomes (e.g., behavior change or literacy) Additionally, the support groups and peer navigation components could not be piloted due to restrictions related to the COVID-19 pandemic.

Once the pilot culminated, a healthy facility director described how there was initially some hesitation in using the new tools but that HCWs were able to meet the demand. This suggests that the integration of new tools to support adherence was both feasible and accepted by providers and AYAHIV clients.

*I remember that when I was doing the first training in which I had to talk about the new instruments, the HCWs were the first to say that “this package is big, it is a lot, and it will take me a lot of time and the consultation is long.” Today we are almost at the end of the pilot, and they are the same HCWs who say that “it cannot end because we are already managing to meet the demand and the adolescents like it.”* —Key informant interview

Based on recommendations from all pilot participants, future implementation should consider increasing privacy in the consultation rooms so that AYAHIV are more comfortable using the CombinADO tools, communicating that wait times may increase as HCWs become familiar with CombinADO tools during consultations, facilitating communication between health facilities engaged in the study to share best practices, and addressing the needs of AYAHIV with low literacy. Prototype-specific recommendations primarily included changes to design elements (e.g., changing colors, adding clarifying language or additional inspirational messaging, or including real people to increase credibility).

### Implementation

In Phase 2, a cluster-randomized control trial will be used to evaluate the effectiveness of the CombinADO intervention in increasing retention in care, ART adherence, and viral suppression at 12 health clinics in Nampula (clinicaltrials.gov: NCT04930367).^30^

## DISCUSSION

Adolescents and young people represent a growing proportion of people living with HIV and present the highest rates of attrition across the HIV care cascade and of AIDS-related mortality.^12^ Few evidence-based interventions have been proven to be effective in achieving viral suppression in this population, and there is an urgent need to design, implement, and test interventions that retain adolescents and young people in care.^12^

The most promising strategies for treatment adherence and retention in HIV care for AYAHIV involve multiple components and innovative interventions that are acceptable, feasible to deliver, and responsive to the unique ART adherence challenges and contextual realities experienced by AYAHIV.^31^ In addition to managing a chronic condition, AYAHIV must navigate physical, social, and emotional changes and the challenges of navigating sexual health, relationships, and emotional well-being; HIV stigma and disclosure; caregiver stress; peer relations; mental health and/or substance use; and medication management.^32^^,^^33^ Multicomponent interventions tend to be bottom-up and community-driven, where communities determine a set of interventions, adopt or adapt a particular combination of interventions that are implemented simultaneously, and evaluate effectiveness in real-world settings.^34^ HCD offers a framework to incite empathy and foster community engagement to ensure that public health services, products, and policies are readily adopted, effective, and innovative.^35^ When used as a participatory method, the HCD process allows for quicker intervention development, dissemination, and implementation of interventions that are acceptable, feasible, desirable, scalable, and sustainable. In contrast to traditional research methods, HCD is less protocol driven and more flexible and responsive to community needs. The investment of greater resources upfront to codesign and tailor interventions for the local context helps to detect key issues for integration of new interventions within existing systems and identifies barriers and facilitators that may affect uptake.^16^^,^^36^

The most promising strategies for treatment adherence and retention in HIV care for AYAHIV involve multiple components and innovative interventions that are acceptable, feasible to deliver, and responsive to the unique ART adherence challenges and contextual realities experienced by AYAHIV.

When used for research, HCD can employ the principles of community-engaged research^37^ and participatory methods by integrating communities into the research team to assist with the refinement of research questions, participant recruitment, data collection, interpretation of findings, intervention development, and evaluation. An interdisciplinary team of stakeholders and the integration of nonacademic researchers that include nonsubject matter experts bring a “beginner's mindset” (i.e., a new set of eyes on some very old problems), which often leads to remarkably insightful findings. The HCD process also embeds researchers into a community, which better enables researchers to garner permissions, facilitate stakeholder buy-in, and build empathy, trust, and credibility.^38^ Furthermore, HCD combines multiple data collection methods (e.g., ethnographic observations and quantitative and qualitative data) to understand the complex system in which individuals reside and interact. Similarly, systems thinking can include qualitative, action-based methodologies that enable stakeholders to examine system components and the dynamic relationship between them, across multiple levels across the socioecological model.^39^ By incorporating systems thinking, HCD seeks to understand the broader context in which individuals are interconnected within societies and considers root causes of a problem and downstream consequences of solutions to complex health problems. Moreover, HCD brings creativity and innovation to traditional public health programming and may be best paired with experts from other disciplines (e.g., community-based participatory research or implementation science) to amplify its value.^35^^,^^36^ HCD can also promote greater intervention uptake by placing community needs at the forefront and may be especially useful in ensuring mass media and interpersonal communication tools remain culturally relevant so that interventions are not only acceptable but also desirable.

This study demonstrates the utility of using HCD to cocreate a multicomponent intervention to promote adherence and retention in care for AYAHIV. Prior studies have found that youth-driven interventions lead to greater structural level changes,^40^ and the use of HCD for youth interventions has successfully been applied to a variety of public health problems including increasing access to sexual and reproductive health for girls and boys, uptake of contraceptives and HIV self-testing, advancing gender equity, development of learning hubs for refugees and displaced children, development of adolescent-friendly health clinics and safe spaces, violence reduction, and diabetes prevention.^41^^–^^44^ Although HCD methodologies can be used with nearly any population, they may be particularly well-suited for youth-driven intervention development. HCD provides a framework for cocreating interventions in collaboration with youth by incorporating lived experiences and provoking empathy and mutual understanding of the root causes of a problem within the larger socioecological environment. HCD methodologies are less formal than traditional qualitative and quantitative research methods; however, they are able to collect “unconventional” data by incorporating interactive, visual, and observational data collection methods that more closely resemble play. For example, data can be collected through role playing in which youth “show” how they interact with products, services, or programs, rather than tell to ensure that youth have real agency in defining the service experience. Furthermore, participants may also be asked to rank cards, sticky notes, photos, and use colors or drawings to express priorities and feelings, rather than use traditional Likert scales. This has several advantages, including quicker data collection, data processing, and hypothesis generation (or refinement) in the moment. This also addresses literacy challenges which enables a wider variety of youth to participate and ensures language and content remain adolescent friendly. When used with youth populations, HCD legitimizes the experiences and opinions of youth, fosters youth empowerment, allows youth to invest in their communities, and addresses power imbalances between youth and adult stakeholders.^40^

When used with youth populations, HCD legitimizes the experiences and opinions of youth, fosters youth empowerment, allows youth to invest in their communities, and addresses power imbalances between youth and adult stakeholders.

### Challenges and Limitations

Although HCD has been used for health care intervention development, relatively little HCD or design thinking has been reported within the academic literature. Instead, HCD projects tend to be reported in the gray literature, lack clear methodology, and use varied language for similar concepts.^45^ Although organizations like Design for Health have tried to bridge the gap between HCD designers and public health practitioners, robust, academic literature on HCD and public health is still an emerging area.^17^ The use of HCD by academic researchers will require training, familiarity with HCD terminology, and the expertise of a design firm, which can be high cost and resource intensive. The HCD approach is not designed to evaluate efficacy or effectiveness but can be rigorously evaluated via randomized control trials. ^30^^,^^46^ Furthermore, the final intervention may have limited generalizability since data are collected via purposive sampling to be contextually specific. However, the intervention may be transferred and inform intervention development in other settings. Despite these limitations, the flexibility and creativity of HCD allow for public health innovation with applications in global health.^47^

## CONCLUSION

Overall, this article demonstrates a creative process embedded in a traditional research study. HCD can serve as a community engagement model by incorporating community values, generating buy-in, and building consensus and collective ownership early on. A small but growing body of evidence shows that HCD, when coupled with other synergistic research methods such as community-based participatory research^31^ or implementation science,^32^ provides a pathway for innovation, shortens the research to practice gap, engages marginalized communities, and integrates novel interventions into existing complex systems. When used as a precursor to implementation science, the HCD process ensures that intervention development (or adaption) is grounded in contextual needs, assumptions are tested early, and that concepts are rapidly iterated upon and refined before resources are dedicated to implementation and scale-up.

## Translation

**Em Português**

**Design centrado no ser humano para inovação em saúde pública: co-design de uma intervenção multicomponente para apoiar os jovens em todo o continuum de cuidados de HIV em Moçambique**

Conclusões principais:

Através do uso do design centrado no ser humano (DCH), foram identificadas oportunidades de intervenção para promover a supressão viral do HIV, melhorar a adesão ao TARV e a retenção nos cuidados de saúde entre AJVHIV. As oportunidades incluíram a promoção de um sentimento de pertença e ligação com outros AJVHIV, a oferta de informação médica contextualmente relevante e adequada ao contexto único da adolescência, a redução do estigma e mitos em torno do HIV e a promoção de um sentido de esperança entre os AJVHIV.

Os componentes da intervenção incluem uma campanha dos media para reduzir o estigma e aumentar a literacia em torno do HIV; um kit de ferramentas para ajudar os provedores a comunicarem e responderem melhor às necessidades dos pacientes AJVHIV; grupos de apoio de pares para melhorar a literacia em torno do HIV, empoderar os jovens e fornecer modelos positivos para pessoas que vivem com HIV; grupos de apoio para pais/cuidadores; e recipientes de comprimidos discretos para promover a adesão à medicação fora de casa.

Implicações principais:

Os profissionais de saúde pública devem ter em consideração a mais valia do DCH apara abrir um caminho para a inovação em saúde pública, reduzir as lacunas entre a pesquisa e a prática, envolver comunidades marginalizadas e integrar novas intervenções em sistemas complexos quando combinado com outros métodos de pesquisa sinérgicos, tais como a ciência de implementação.

Resumo:

Os adolescentes e jovens representam uma proporção crescente de pessoas vivendo com HIV (AJVHIV), e há uma necessidade urgente de projetar, implementar e testar intervenções que mantenham os AJVHIV nos cuidados de saúde. Através de uma abordagem de design centrado no ser humano (DCH), co-projetamos o CombinADO, uma intervenção para promover a supressão viral do HIV, melhorar a adesão ao TARV e a retenção nos cuidados de saúde entre AJVHIV em Nampula, Moçambique. O processo de DCH envolve pesquisa formativa com AJVHIV, profissionais de saúde, pais/cuidadores e especialistas em HIV com adolescentes, síntese das descobertas para gerar orientações para a acção, idealização e prototipagem de componentes de intervenção e um estudo piloto para avaliar a viabilidade, aceitabilidade e utilização das componentes da intervenção.

O CombinADO promove a adesão ao TARV ao promover a ligação e a pertença entre pares, fornece conhecimento médico acessível; desmistifica e desestigmatiza o HIV, e cultiva um sentido de esperança entre os AJVHIV. Os protótipos bem sucedidos incluíram uma campanha dos média para reduzir o estigma e aumentar a literacia entre torno do HIV; uma brochura para ajudar os profissionais de saúde a comunicarem e atenderem melhor às necessidades exclusivas dos AJVHIV; grupos de apoio para AJVHIV para melhorar a literacia sobre o HIV, capacitar os AJVHIV e fornecer modelos positivos de pessoas vivendo com HIV; grupos de apoio para pais/cuidadores; e recipientes de comprimidos discretos para facilitar a adesão à medicação fora de casa. Na próxima fase, a eficácia do CombinaADO na retenção nos cuidados de saúde, adesão ao TARV e supressão viral do HIV será avaliada através de um estudo de controle randomizado por cluster.

Neste artigo, demonstramos a utilidade de usar o DCH para co-criar uma intervenção multicomponente. Também discutimos como a metodologia DCH enriquece os métodos participativos e o envolvimento da comunidade. Este processo é ilustrado pelo desenvolvimento do CombinADO através de uma intervenção dirigida por adolescentes e jovens, pela promoção do empoderamento dos jovens, pela abordagem dos desequilíbrios de poder entre os actores jovens e adultos e garantindo o uso consistente de uma linguagem e o conteúdos amigáveis aos adolescentes e jovens.

**Peer Reviewed**

**First published online:** April 21, 2022.
